# Genetic Susceptibility to Glomerulonephritis in Children: Analysis of Structural Kidney Genes and Immune System Genes

**DOI:** 10.3390/jcm14145119

**Published:** 2025-07-18

**Authors:** Marina Peric, Aleksandra Anicin, Marija Brankovic, Natasa Stajic, Jovana Putnik, Aleksandra Paripovic, Milena Jankovic, Ivo Bozovic, Vladimir Perovic, Ivana Novakovic, Vladislav Vukomanovic, Emina Milosevic

**Affiliations:** 1 Mother and Child Healthcare Institute of Serbia “Dr. Vukan Cupic”, 11070 Belgrade, Serbia; natalijast2003@yahoo.com (N.S.); putnik.jovana@gmail.com (J.P.); liki_74@yahoo.com (A.P.); vvukomanovicdr@gmail.com (V.V.); 2 Institute of Microbiology and Immunology, Faculty of Medicine, University of Belgrade, 11000 Belgrade, Serbia; aleksandra.anicin@med.bg.ac.rs (A.A.); perovic.vladimir@gmail.com (V.P.); emina.milosevic@med.bg.ac.rs (E.M.); 3 Department for Clinical Genetics, University Children’s Hospital, 11000 Belgrade, Serbia; mara.brankovic@gmail.com; 4 Faculty of Medicine, University of Belgrade, 11000 Belgrade, Serbia; 5 Neurology Clinic, University Clinical Center of Serbia, 11000 Belgrade, Serbia; milena.jankovic.82@gmail.com (M.J.); ivo.bozovic20@gmail.com (I.B.); 6 Institute for Human Genetics, Faculty of Medicine, University of Belgrade, 11000 Belgrade, Serbia; tetaana61@yahoo.com

**Keywords:** glomerulonephritis, cytokines, single nucleotide polymorphism, tumor necrosis factor, *TNF* gene

## Abstract

**Background/Objectives**: Glomerulonephritis (GNs) is a heterogeneous group of inflammatory kidney diseases. Novel genetic methods have revealed some disease-causing and susceptibility genes underlying primary and secondary GNs. We aimed to investigate the presence of the single nucleotide polymorphisms (SNPs) rs12917707, found in the *UMOD* gene, and rs17319721, found in the *SHROOM3* gene, as well as different polymorphisms in immune system genes in a group of children with GN. **Method**: The study included 71 children with GN (40 with primary and 31 with secondary GN) and 119 healthy children (HC). SNPs of the *UMOD* (rs12917707), *SHROOM3* (rs17319721), *IL10* (rs1800871 and rs3024505), *IL6* (rs1800795), *IL12B* (rs3212227), *IL23R* (rs11209026 and rs1800896), and *TNF* (rs361525 and rs1800629) genes were genotyped. **Results**: The median age of the patients was 8 years at the onset of GN and 14 years at sampling. Allele A for rs1800629 in the *TNF* gene was more common in patients with GN in comparison to HCs (*p* = 0.009), followed by the difference in genotype distributions (*p* = 0.021), where AA and GA genotypes were more prevalent in patients. We found a statistically significant difference in haplotype distributions between patients and HCs for TNF, with GN patients having the GGAG haplotype more frequently and HCs having GGGG (*p* < 0.05). No correlation between the investigated SNPs and patient clinical characteristics (disease onset, primary or secondary GN, severity of disease, occurrence of remission, and presence of hypertension) was observed. **Conclusions**: An association between the *TNF* gene and different types of GN was noticed in children with GN. This may help us to understand the pathogenesis of these disorders and develop new treatments to cover the unmet needs of children with GN.

## 1. Introduction

Glomeruloneprhitides (GNs) are a heterogeneous group of inflammatory diseases, which can be primary if they only affect the kidneys and secondary if the kidneys are damaged due to the presence of another systemic disease. There are several main subtypes of primary GNs: immunoglobulin A nephropathy (IgAN), membranous nephropathy (MN), minimal change disease (MCD), focal segmental glomerulosclerosis (FSGS), and membranoproliferative GN (MPGN). On the other hand, in secondary GNs, kidneys are mostly affected due to systemic lupus erythematosus (SLE) (lupus nephritis—LN) and Henoch–Schoenlein purpura (HSP) [1,2,3]. Although their clinical presentations vary, these diseases share overlapping immunopathogenic mechanisms that often involve dysregulation of immune responses and glomerular structural damage.

Genome-wide association studies (GWAS), whole exome sequencing (WES), and whole genome sequencing (WGS) revealed some disease-causing and susceptibility genes underlying primary and secondary GNs. The occurrence of IgA nephropathy is mostly associated with genes encoding proteins of the immune system [3,4,5,6]. These genes are associated with the pathogenesis of MN as well, including polymorphic variants in the inflammatory cytokines such as the tumor necrosis factor (*TNF*), interleukin (IL)-4 (*IL4)*, and *IL10* genes [7]. HLA polymorphisms and genes important for interferon signaling are associated with LN [8]. The most significant association of HSP was observed with the HLA locus [9,10]. Polymorphisms in genes encoding *IL18* and other immune system genes were also identified as a risk factor for HSP [11,12]. Taken together, these findings suggest that specific genetic variants in immune-related pathways may predispose individuals to different forms of GN while influencing disease severity and outcome.

The factors leading to the progression of GN to chronic kidney disease (CKD) are not fully understood. The genes that define CKD generally do not overlap with those identified as susceptibility genes for specific kidney diseases [13]. Previous research has identified two distinct groups of genes in which certain variants may confer susceptibility to CKD [14,15]. The first group contains the genes that encode kidney proteins, where polymorphisms in the *UMOD* and *SHROOM3* genes are consistently identified [16,17,18,19]. UMOD encodes uromodulin, the most abundant protein in normal urine, which plays a role in tubular function and innate immunity, while SHROOM3 is involved in maintaining the cytoskeletal architecture necessary for glomerular filtration barrier integrity [20,21,22]. Variants in these genes may contribute to progressive structural damage in the kidney.

The second group of polymorphisms is found in genes encoding proteins of the immune system. The polymorphisms rs1800871 and rs1800896 in the gene encoding *IL10* can be associated with the development of complications of kidney diseases in children [23]. IL10 and IL4 play key roles in modulating inflammatory responses, and the altered cytokine expression caused by these SNPs may result in a sustained inflammatory milieu in glomeruli. TGFB1 encodes transforming growth factor beta 1, a central mediator of fibrosis and extracellular matrix deposition, making its variants biologically relevant for the progression of CKD. The association of gene polymorphisms in other interleukins and growth factors with the onset of CKD has also been described, including polymorphism rs8179190 in the *IL4* gene, as well as rs1800469, rs1800471, and rs1800470 in the *TGFB1* gene [24]. TNF is a pleiotropic cytokine implicated in the regulation of cell proliferation, apoptosis, differentiation, coagulation, and lipid metabolism and is involved in a variety of diseases, including insulin resistance, carcinoma, and autoimmune diseases [25,26,27,28]. Additionally, the severity of SLE has been associated with increased levels of TNF [29]. These increased levels are associated with disease activity and several systemic symptoms, including SLE-related cardiovascular illness and lupus nephritis [30]. Further, the TNF pathway has also been implicated in the pathogenesis of other types of GN, including FSGS and minimal changes kidney disease (MCD). In these diseases, both mesangial cells and podocytes have been found to increase TNF production through autocrine mechanisms, leading to the upregulation of TNF receptors [31]. These elevated TNF pathway markers have been observed to contribute significantly to renal impairment [32]. In this context, the selection of these specific SNPs allows us to explore their genetic contributions to both immune dysregulation and structural kidney damage in glomerular diseases.

The aim of this study was to investigate the presence of polymorphism rs12917707 in the *UMOD* gene, rs17319721 in the *SHROOM3* gene, rs1800871 and rs3024505 in the *IL10* gene, rs1800795 in the *IL6* gene, rs3212227 in the *IL12B* gene, rs11209026 and rs1800896 in the *IL23R* gene, and rs361525 and rs1800629 in the *TNF* gene in a group of children from Serbia with GN.

## 2. Patients and Methods

This research included patients with GNs from the Institute for Mother and Child Healthcare of Serbia “Dr. Vukan Cupic”. Patients with primary or secondary GNs (due to SLE, HSP, and ANCA vasculitis) were consecutively collected from the Inpatient and Outpatient Unit of the Institute from 1 March 2021 to 1 March 2022. This study was approved by the Ethical Committee of the Faculty of Medicine, University of Belgrade (No. 1322/VII-48 from 29 July 2020), and informed consent was obtained from all patients and/or their parents/caregivers before blood sampling.

Diagnosis was established based on the clinical presentation, laboratory results, and, in the majority of cases, histopathology findings. We excluded patients with non-inflammatory glomerular disease, patients who had a positive family history of kidney disease or proven genetic kidney disease, and those who had GN and another significant disease unrelated to GN. A total of 85 patients were primarily seen during the observed period. However, 14 patients were excluded for various reasons: 3 were excluded due to their parents’ refusal to allow their child to participate in the study, 10 patients had SLE and HSP without proven kidney involvement, and 1 patient was excluded after genetic confirmation of atypical hemolytic uremic syndrome. The final number of patients included in the research was 71. The prevalence of SNP alleles and genotypes was also analyzed in a cohort of 119 healthy children collected as part of the project “Yugoslav study of precursors of atherosclerosis in school children”. This cohort consisted of 60 females and 59 males aged 14 years at the time of sampling.

Genomic DNA was extracted from patients’ peripheral blood using a PureLink^®^ Genomic DNA Mini Kit (Invitrogen, Waltham, MA, USA) and from healthy controls using the salting out method [33]. The genotyping of SNPs in the *UMOD* (rs12917707) and *SHROOM3* (rs17319721) genes was conducted in the laboratory for molecular and genetic diagnostics of neurological diseases at the Neurology Clinic of the University Clinical Center of Serbia. Genotyping of the SNPs in the *IL10* (rs1800871 and rs3024505), *IL6* (rs1800795), *IL12B* (rs3212227), *IL23R* (rs11209026 and rs1800896), and *TNF* (rs361525 and rs1800629) genes was performed at the Institute of Microbiology and Immunology, University of Belgrade—Faculty of Medicine, Serbia. Real-time polymerase chain reaction (RT-PCR) SNP analysis was performed using TaqMan genotyping assays (ThermoFisher Scientific, Foster City, CA, USA) on the ABI Prism 7500 Fast Real-Time PCR System (Applied Biosystems, Waltham, MA, USA). The obtained results were processed using the 7500 Software program (Applied Biosystems, Waltham, MA, USA).

Categorical data (nominal and ordinal) were presented as an absolute number and a proportion. The Kolmogorov–Smirnov test was used to test for Gaussian distribution of the numerical data. Non-parametric data were presented as a median and an interquartile range, while parametric were presented as a mean with standard deviation. All results were tested for Hardy–Weinberg equilibrium. *p* values that were less than 0.05 were considered statistically significant. The distributions of allele, genotype, and haplotype frequencies between cases and controls were compared via Pearson’s chi-squared test or Fisher’s exact test, where appropriate. Two-tailed *p* values, odds ratios (OR), and 95% confidence intervals (CI) were calculated. The estimation of haplotype frequencies was carried out via the Expectation–Maximization algorithm using Arlequin 3.5.1.3 [34]. *p* values less than 0.05 were considered significant. Associations between different clinical features of the disease and allele, genotype, allele carrier, and haplotype distribution were assessed using the chi-squared test. Due to the small sample size in different subgroups of GN, there was not enough statistical power to test for SNP distribution in these subgroups. However, we were able to compare frequencies in primary vs. secondary GN. The IBM Statistical Package for Social Sciences, version 20 (IBM SPSS, Chicago, IL, USA), was used for statistical analyses.

## 3. Results

Primary glomerulonephritis was diagnosed in 40 (56.3%) cases from our cohort, and secondary in 31 (43.7%). The median age at the onset of the disease was 8 years, and at sampling it was 14 years. The majority of patients were treated with steroids and/or non-steroid immunosuppressants. After treatment, complete remission without any therapy was achieved in 39 (54.9%) children during a median follow-up of 6.5 years. The main sociodemographic and clinical features of the investigated patients are presented in Table 1. Additional laboratory data at the time of diagnosis and at sampling are presented in Table 2.

All analyzed SNPs were in Hardy–Weinberg equilibrium in both patients and HCs, except for rs12917707 in the *UMOD* gene in the patient group and rs3212227 in the *IL12B* gene in the HC group. Allele A for rs1800629 in the *TNF* gene was more common in patients with GN in comparison to HCs (*p* = 0.009). This pattern was also observed for genotype frequencies, with GA and AA genotypes being more common in our patients than in our HCs (*p* = 0.021) (Table 3). No other differences were observed between the patients and HCs regarding allele frequencies and genotype frequencies in the examined genes.

Since three SNPs were analyzed in the *IL10* gene, we performed a haplotype analysis of this gene. Two rare haplotypes (CGGTGG, N = 1 and TGATAA, N = 1) were omitted from the analysis. The frequencies of *IL10* haplotypes were comparable in GN patients and HCs. We also analyzed *TNF* haplotypes since two SNPs were assessed in this gene. A rare haplotype (GAAG, n = 1) was omitted from the analysis. We found statistically significant differences in *TNF* haplotype distribution between patients and HCs (Table 4).

Due to the small sample size in different subgroups of GN, there was not enough statistical power to test for SNP distribution in these subgroups. However, we were able to compare frequencies in primary vs. secondary GN. There was no significant difference in allele, genotype, allele carrier, and haplotype distribution between patients with primary vs. secondary GN. Furthermore, we analyzed the associations between the frequency distribution of the alleles, genotypes, allele carriers, and haplotypes (where applicable) of the assessed genes and the following clinical characteristics: disease onset, severity of the disease reflected as therapy requirement, occurrence of remission, and presence of hypertension. We used the median of 8 years as our cut-off point and did not find any statistically significant differences with regards to the age at onset of GN and SNP variations. Similarly, there was no significant difference in allele, genotype, allele carrier, and haplotype distribution between patients (I) who did not require therapy, those who underwent corticosteroid treatment, those who needed immunosuppressive or rituximab therapy, and those who experienced (II) remission or developed (III) hypertension.

## 4. Discussion

The main finding of our research is that allele A for rs1800629 in the *TNF* gene was more common in patients with GN in comparison to HCs, and that difference was also observed in the genotype distribution between GN patients and HCs. This finding suggests that rs1800629 might be involved in the aggravation of glomerular inflammation in children. As noted previously, TNF is one of the principal immune system pro-inflammatory cytokines and is known to control inflammation, and it is believed to take part in the development of many autoimmune and inflammatory disorders, including GN. Accordingly, polymorphisms in the genes that regulate the production of TNF might be essential determinants of the risk and severity of the disease.

Our observation that the A allele of SNP rs1800629 in the *TNF* gene was more frequent in children with GN is in accordance with the fact that it is known to influence gene expression and is linked to various infectious and autoimmune diseases [35]. The G allele of SNP rs1800629 is common, while the A allele is rare. Allele A has been associated with higher spontaneous or stimulated expression levels of TNF, and it has been shown that individuals carrying the GA genotype have higher TNF messenger ribonucleic acid (mRNA) and serum TNF protein levels than individuals with the GG genotype [36]. Increased expression of TNF has been demonstrated in vivo in several animal models of GN [37]. In accordance with this is our finding of an association between TNF SNPs and GN, regardless of the GN type (primary or secondary). Regardless of the underlying cause, these results collectively imply that rs1800629 allele A may be a functional variant that promotes sustained TNF activity, facilitating persistent glomerular inflammation and immune-mediated damage. This is consistent with established GN pathogenesis mechanisms in which TNF is essential for leukocyte recruitment, endothelial activation, and glomerular cytokine amplification loops. Our finding suggests that both primary and secondary GN, with their multiple subtypes, may share some similar pathogenetic pathways.

We did not observe an association between TNF SNPs and the disease features of our GN patients, including the severity of the disease, the occurrence of remission, and the presence of complications, i.e., hypertension. This could imply that although TNF polymorphisms may affect the onset of or susceptibility to disease, they do not always predict the clinical course or results, which are probably influenced by a mix of genetic, epigenetic, environmental, and therapeutic factors. To elucidate this relationship, longer-term research and larger cohorts are required. Accordingly, the A allele at position rs1800629 in the *TNF* gene remains controversial regarding its effect on the development of CKD. Additionally, G/G genotype and the G allele (rs1800629) were more common in CKD patients [38]. Oppositely, a study of a North Indian cohort reported that the GA haplotype in rs1800629 had a significant association with end-stage renal disease (ESRD) and may be a strong predisposing risk factor for this condition [39]. A meta-analysis performed by Sookoian et al. in 2005 demonstrated that the presence of the A allele was positively correlated with the occurrence of obesity, hypertension, and elevated insulin levels [40]. We did not find a statistically significant correlation between body mass index and this SNP. However, given the young age of our cohort, metabolic comorbidities may be less common or not yet apparent, which could account for this lack of association. The metabolic effects of this genetic variation may be better captured in future research involving teenagers and young adults.

Finding associations between SNPs in the *TNF* gene and GN might also have therapeutic implications. It is known that in patients with Behçet syndrome (BS) [41], the frequency of the rs1800629 wild-type GG genotype is higher in cases that respond to therapy compared to the GA genotype, suggesting a possible role of the SNP-containing genotype in affecting the anti-TNF drug response [41]. Our results suggest that the GA allele may be a risk factor of GN development in children, while data from the literature add further information suggesting GA allele to be a factor associated with a poor response to potential treatment with TNF inhibitors. Still, we should be very cautious about TNF treatment since we do not know whether this or other SNPs may carry the risk of worsening GN or producing more side effects. TNF inhibitors revolutionized the treatment of multiple autoimmune conditions such as Crohn’s disease, rheumatoid arthritis, psoriasis, psoriatic arthritis, ankylosing spondylitis, and juvenile idiopathic arthritis [42,43]. On the other hand, these drugs have been shown to induce antinuclear antibodies, anti-dsDNA antibodies, and antiphospholipid antibodies, among others [44]. Several autoimmune diseases have been associated with TNF inhibitor exposure, including vasculitis, SLE, and psoriasis [45]. A similar finding has been reported regarding the development of GN in patients treated with anti-TNF drugs [46,47,48]. Thus, the role of these drugs is uncertain in GN, and may be beneficial in certain subtypes, possibly depending on the *TNF* SNPs. Our results lend support to the idea that personalized medicine strategies that consider TNF genotype data could be used in the future to find patients who would benefit most from anti-TNF treatment while preventing possible autoimmune side effects in those who are genetically predisposed. How rs1800629 and its related variants affect GN’s response to TNF inhibitors may be the subject of future pharmacogenomic research.

In the present study we identified an association between the *TNF* haplotype GGAG (rs1800629 and rs361525) and GN. One the other hand, GGGG was more abundant in HCs, in consistency with the fact that the GG genotype is a low TNF producer. This indicates a possible haplotype-based impact on the regulation of the TNF gene, perhaps as a result of the combined effects of both SNPs on transcriptional activity. Even though individual SNPs might only have slight effects, when several regulatory variants work together, haplotypes can represent a more accurate genetic predisposition. One study analyzed the associations of rs361525 and rs1800629 in the *TNF* gene in CKD patients in a Mexican population. They did not observe an association between any haplotype combination of these SNPs and CKD [49]. To the best of our knowledge, no other study has investigated the associations between these haplotypes and CKD.

The allele and genotype distributions for the IL10, IL6, IL23R, and IL12B genes, as well as the haplotype distribution for IL10, did not differ statistically significantly from one another. We found no association between these genes and GN, despite the fact that they are involved in immune regulation and have been linked to a number of autoimmune and inflammatory diseases. This could be because of population-specific genetic variation (children from Serbia), the small sample size, or the possibility that these variants contribute to the severity or progression of the disease rather than its initial pathogenesis. For instance, polymorphisms in the *IL10* gene can be associated with complications of kidney diseases in children [23]. Thus, sampling later in the course of the disease may better capture patients with chronic complications and their relationship with *IL10* SNPs.

No differences were observed between patients and HCs regarding allele frequencies and genotype frequencies in structural kidney genes (*UMOD* and *SHROOM3*). The reason could be that we included all patients with GN, both acute and those that progressed to chronic disease, and the *UMOD* and *SHROOM3* genes are mostly associated with CKD [14,15,16,17,18,19,20]. However, even our sub-analysis did not show an association between these genes and a worse prognosis of GN. The main reason may be the small number of analyzed patients. It is also plausible that these genes exert their pathogenic influence later in the disease course or in response to chronic injury, which may not be captured in cross-sectional pediatric cohorts. Sampling later in the disease course, conducting studies with a longitudinal design, or using larger datasets stratified by CKD stage may make it easier to detect subtle genetic contributions from UMOD and SHROOM3 in GN.

The main limitations of this research are the relatively small number of patients included and the heterogeneous diagnoses. Unfortunately, there was not enough statistical power to test for SNP distribution among patients with different types of GN. However, this is the first study conducted in Serbia and the surrounding countries investigating genetic susceptibility to GNs in children. This emphasizes the importance of regionally tailored genomic research, which can uncover population-specific risk factors not apparent in large, multi-ethnic consortia. Additionally, it establishes the foundation for future cooperative genetic epidemiology initiatives in Southeast Europe.

In conclusion, allele A for rs1800629 in the *TNF* gene was more common in patients with GN in comparison to HCs. In line with this result, GA and AA, genotypes with presumed higher TNF production, were overrepresented among the patients. Moreover, we found statistically significant differences in haplotype distribution between patients and HCs in terms of TNF, with GN patients having the GGAG haplotype more frequently and HCs primarily having the GGGG haplotype. However, due to the small number of patients per subtype of GN, we did not conduct separate SNP association analyses with each clinical entity. We speculate that the association between *TNF* SNPs and different types of GN in children might contribute to the understanding of the pathogenesis of these disorders and the development of new treatments.

## Figures and Tables

**Table 1:** Main sociodemographic and clinical features of investigated children with GN (N = 71).

Feature	N (%) or Median (IQR)
Type of glomerulonephritis	
Primary	40 (56.3%)
Steroid-sensitive nephrotic syndrome	12 (16.9%)
IgA nephropathy	10 (14.1%)
IgM nephropathy	7 (9.9%)
Membranoproliferative glomerulonephritis	5 (7.0%)
Focal segmental glomerulosclerosis	3 (4.2%)
Membranous glomerulonephritis	1 (1.4%)
Anti-glomerular basement membrane glomerulonephritis	1 (1.4%)
Rapid progressive glomerulonephritis	1 (1.4%)
Secondary	31 (43.7%)
Lupus nephritis	19 (26.8%)
Henoch–Schoenlein purpura nephritis	10 (14.1%)
Glomerulonephritis in ANCA vasculitis	2 (2.8%)
Gender	
Male	33 (46.5%)
Female	38 (53.5%)
Age at onset (years)	8 (4–13)
Diagnostic delay (months)	1 (0–2)
Estimated glomerular filtration rate (mL/min/1.73 m^2^) at diagnosis	133 (103–158)
<90%	12 (16.9%)
Duration of disease before sampling (months)	67 (38–103)
Age at sampling (years)	14 (7–17)
Therapy during disease course	
None	7 (9.9%)
Corticosteroid	23 (32.4%)
Corticosteroid and non-steroid immunosuppressant	34 (47.9%)
Corticosteroid, non-steroid immunosuppressant, and rituximab	7 (9.9%)
Complete remission	
Yes	39 (54.9%)
No	32 (45.1%)
Time from therapy initiation to complete remission (years)	6.5 (5–11.5)
Dialysis	
No	67 (94.4%)
Acute	2 (2.8%)
Chronic	2 (2.8%)
Hypertension at sampling	
Yes	32 (45.1%)
No	39 (54.9%)
Estimated glomerular filtration rate (mL/min/1.73 m^2^) at sampling	148 (126–163.5)
<90%	7 (9.9%)

**Table 2:** Laboratory findings of children with GN at time of diagnosis and at time of blood sampling (N = 71).

Characteristic	At the Time of Diagnosis (N (%) or Mean (Standard Deviation) or Median (Interquartile Range))	At the Time of Sampling (N (%) or Mean (Standard Deviation) or Median (Interquartile Range))
C-reactive protein (mg/L)	3.5 (0.75–10.7)	0.5 (0.3–2.15)
Erythrocyte sedimentation rate (mm/h)	60 (20–80)	8 (5–18.5)
Blood hemoglobin (g/L)	121.7 (17.2)	131.1 (15.9)
Glycemia (mmol/L)	4.8 (0.9)	4.8 (0.4)
Urea (mmol/L)	4.5 (3.7–6.1)	4.1 (3.5–5.2)
Creatinine (umol/L)	50 (35.5–70)	50 (39–69)
Protein (g/L)	62.0 (13.6)	67.4 (8.6)
Albumin (g/L)	37 (26–43)	45 (41.5–46)
Sodium (mmol/L)	137.6 (2.8)	138.8 (2.8)
Potassium (mmol/L)	4.3 (0.8)	4.3 (0.4)
Chloride (mmol/L)	101.6 (3.3)	102.0 (2.7)
Calcium (mmol/L)	2.12 (0.56)	2.36 (0.33)
Magnesium (mmol/L)	0.81 (0.19)	0.82 (0.13)
Phosphorus (mmol/L)	1.44 (0.42)	1.45 (0.34)
Bicarbonate (mmol/L)	22.4 (2.8)	23.6 (2.1)
Triglycerides (mmol/L)	1.5 (0.89–2.29)	1.0 (0.61–1.56)
Total cholesterol (mmol/L)	5.04 (4.18–7.57)	4.7 (4.14–5.74)
HDL cholesterol (mmol/L)	1.34 (1.02–1.84)	1.79 (1.2–2.0)
LDL cholesterol (mmol/L)	2.68 (2.16–4.7)	2.94 (2.26–3.42)
Hematuria	50 (70.4%)	18 (25.4%)
Proteinuria	35 (49.3%)	8 (11.3%)
24 h proteinuria (mg/day)	942 (263.5–1877.5)	135.5 (81–304.5)

**Table 3:** Allele and genotype frequencies of SNPs in controls and patients with GN.

Gene (SNP)	HC (N = 119) N (%)	GN Patients (N = 71) N (%)	*p* Value	OR (95% CI)
***IL10* (rs1800896)**				
Allele	238	142		
G	102 (42.9)	64 (45.1)	0.671	1.094 (0.719–1.663)
A	136 (57.1)	78 (54.9)		
Genotype	119	71		
GG	20 (17.1)	12 (16.9)	0.806	1.108 (0.506–2.424)
GA	62 (53.0)	40 (56.3)		0.843 (0.466–1.522)
AA	37 (31.6)	19 (26.8)		1.220 (0.635–2.343)
***IL10* (rs1800871)**				
Allele	238	142		
C	179 (75.2)	114 (80.3)	0.254	1.342 (0.807–2.229)
T	59 (24.8)	28 (19.7)		
Genotype	119	71		
CC	66 (56.4)	46 (64.8)	0.434 ^§^	1.477 (0.805–2.709)
CT	47 (40.2)	22 (31.0)		0.687 (0.368–1.282)
TT	6 (5.1)	3 (4.2)		0.831 (0.201–3.431)
***IL10* (rs3024505)**				
Allele	236	142		
G	200 (84.8)	120 (84.5)	1.000	0.982 (0.552–1.747)
A	36 (15.2)	22 (15.5)		
Genotype	118	71		
GG	83 (70.9)	52 (73.2)	0.217^§^	1.154 (0.598–2.227)
GA	34 (29.1)	16 (22.5)		0.718 (0.362–1.425)
AA	1 (0.8)	3 (4.2)		5.161 (0.526–50.609)
***TNF* (rs1800629)**				
Allele	238	142		
G	215 (90.3)	115 (81.0)	**0.009**	0.455 (0.249–0.830)
A	23 (9.7)	27 (19.0)		
Genotype	119	71		
GG	97 (82.9)	46 (64.8)	**0.021** ^§^	0.417 (0.213–0.817)
GA	21 (17.9)	23 (32.4)		2.236 (1.127–4.435)
AA	1 (0.8)	2 (2.8)		3.420 (0.304–38.418)
***TNF* (rs361525)**				
Allele	238	142		
G	234 (98.3)	137 (96.5)	0.303 ^§^	0.468 (0.124–1.773)
A	4 (1.7)	5 (3.5)		
Genotype	119	71		
GG	115 (98.3)	66 (93.0)	0.298 ^§^	0.459 (0.119–1.769)
GA	4 (3.4)	5 (7.0)		2.178 (0.565–8.394)
AA	0 (0)	0 (0)		NA
***IL6* (rs1800795)**				
Allele	238	142		
G	156 (65.6)	93 (65.5)	1.000	0.997 (0.644–1.544)
C	82 (34.4)	49 (34.5)		
Genotype	119	71		
GG	52 (44.4)	31 (43.7)	1.000	0.998 (0.552–1.806)
GC	52 (44.4)	31 (43.7)		0.998 (0.552–1.806)
CC	15 (12.8)	9 (12.7)		1.006 (0.415–2.436)
***IL12B* (rs3212227)**				
Allele	234	142		
T	177 (75.6)	103 (72.5)	0.517	0.850 (0.529–1.366)
G	57 (24.4)	39 (27.5)		
Genotype	117	71		
TT	71 (60.7)	35 (49.3)	0.057 ^§^	0.629 (0.347–1.142)
TG	35 (29.9)	33 (46.5)		2.034 (1.103–3.750)
GG	11 (9.4)	3 (4.2)		0.425 (0.114–1.579)
***IL23R* (rs11209026)**				
Allele	238	140		
G	227 (95.4)	135 (96.4)	0.624 ^§^	0.308 (0.445–3.846)
A	11 (4.6)	5 (3.6)		
Genotype	119	70		
GG	108 (92.3)	65 (92.9)	0.788 ^§^	1.324 (0.440–3.982)
GA	11 (9.4)	5 (7.1)		0.755 (0.251–2.271)
AA	0 (0)	0 (0)		NA
***UMOD* (rs12917707)**				
Allele	222	140		
G	178 (80.2)	114 (81.4)	0.764	1.084 (0.632–1.857)
T	44 (19.8)	26 (18.6)		
Genotype	111	70		
GG	73 (65.8)	49 (70.0)	0.636 ^§^	1.214 (0.637–2.313)
GT	32 (28.8)	16 (22.9)		0.731 (0.366–1.462)
TT	6 (5.4)	5 (7.1)		1.346 (0.395–4.589)
***SHROOM3* (rs17319721)**				
Allele	224	142		
A	90 (40.2)	57 (40.14)	1.000	0.998 (0.650–1.533)
G	134 (59.8)	85 (59.86)		
Genotype	112	71		
AA	17 (15.2)	14 (19.7)	0.456	1.372 (0.629–2.993)
AG	56 (50.0)	29 (40.8)		0.690 (0.378–1.259)
GG	39 (34.8)	28 (39.4)		1.218 (0.659–2.253)

*p* values were calculated using the chi-squared test, except for §, where Fisher’s exact test was used. HC, healthy control subjects; GN, glomerulonephritis; OR, odds ratio; CI, confidence interval; NA, not applicable. Statistically significant differences are given in bold.

**Table 4:** Haplotype frequencies for *TNF* in HCs and patients with GN.

*TNF* Haplotypes	HC ^#^ (N = 118) N (%)	GN Patients (N = 70) N (%)	*p* Value	OR (95% CI)
**GGGG**	92 (77.9)	42 (60.0)	**0.008**	0.424 (0.222–0.809)
**GGGA**	4 (3.4)	4 (5.7)	0.473 ^§^	1.727 (0.418–7.136)
**GGAG**	21 (17.8)	22 (31.4)	**0.031**	2.117 (1.061–4.224)
**AGAG**	1 (0.8)	2 (2.8)	0.556 ^§^	3.441 (0.306–38.661)
Total	236	140		

Haplotype analysis was inferred for *TNF* rs1800629 and rs361525. HC, healthy control subjects; GN, glomerulonephritis; OR, odds ratio; CI, confidence interval. *p* values were calculated using the chi-squared test, except for ^§^, where Fisher’s exact test was used; ^#^ *p* values were not calculated for haplotypes with a frequency of less than 1% (i.e., the GAAG genotype, N = 1 in patients). Statistically significant differences are given in bold.

## Data Availability

All the relevant data have been provided in the manuscript and are available from the corresponding author upon reasonable request.
